# Complex postbreeding molt strategies in a songbird migrating along the East Asian Flyway, the Pallas’s Grasshopper Warbler *Locustella certhiola*

**DOI:** 10.1002/ece3.7098

**Published:** 2020-12-21

**Authors:** Hans‐Jürgen Eilts, Nele Feuerbach, Philip D. Round, Oleg Bourski, John Allcock, Paul Leader, Batmunkh Davaasuren, Tuvshinjargal Erdenechimeg, Jong‐Gil Park, Wieland Heim

**Affiliations:** ^1^ Berlin Germany; ^2^ Department of Biology Faculty of Science Mahidol University Bangkok Thailand; ^3^ A.N. Severtsov Institute of Ecology and Evolution Russian Academy of Sciences Moscow Russia; ^4^ School of Biological Sciences Kadoorie Biological Sciences Building, The University of Hong Kong Hong Kong SAR China; ^5^ c/o aec Limited Hong Kong SAR China; ^6^ Wildlife Science and Conservation Center Ulaanbaatar Mongolia; ^7^ Bird Research Center Korea National Park Research Institute Korea National Park Service Sinan‐gun Korea; ^8^ Institute of Landscape Ecology Münster University Münster Germany

**Keywords:** divergent primary molt, fueling, molt migration, molt strategy, postbreeding molt, stopover

## Abstract

Molt strategies have received relatively little attention in current ornithology, and knowledge concerning the evolution, variability and extent of molt is sparse in many bird species. This is especially true for East Asian *Locustella* species where assumptions on molt patterns are based on incomplete information. We provide evidence indicating a complex postbreeding molt strategy and variable molt extent among the Pallas's Grasshopper Warbler *Locustella certhiola*, based on data from six ringing sites situated along its flyway from the breeding grounds to the wintering areas. Detailed study revealed for the first time that in most individuals wing feather molt proceeds from the center both toward the body and the wing‐tip, a molt pattern known as divergent molt (which is rare among Palearctic passerines). In the Russian Far East, where both breeding birds and passage migrants occur, a third of the adult birds were molting in late summer. In Central Siberia, at the northwestern limit of its distribution, adult individuals commenced their primary molt partly divergently and partly with unknown sequence. During migration in Mongolia, only descendantly (i.e., from the body toward the wing‐tip) molting birds were observed, while further south in Korea, Hong Kong, and Thailand the proportion of potential eccentric and divergent feather renewal was not identifiable since the renewed feathers were already fully grown as expected. We found an increase in the mean number of molted primaries during the progress of the autumn migration. Moderate body mass levels and low‐fat and muscle scores were observed in molting adult birds, without any remarkable increase in the later season. According to optimality models, we suggest that an extremely short season of high food abundance in tall grass habitats and a largely overland route allow autumn migration with low fuel loads combined with molt migration in at least a part of the population. This study highlights the importance of further studying molt strategy as well as stopover behavior decisions and the trade‐offs among migratory birds that are now facing a panoply of anthropogenic threats along their flyways.

## Graphical abstract

We analyze molt patterns in a little‐known passerine, the Pallas's Grasshopper Warbler *Locustella certhiola*, by using data from multiple sources and sites. We found complex and variable partial postbreeding molt strategies, including evidence for the rarely described divergent primary molt.

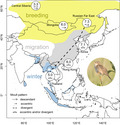

## 1 INTRODUCTION

Annual cycles of birds comprise a series of energy‐ and time‐demanding phenological processes (life‐history stages; Wingfield, 2005): namely breeding, migration, and molt. Feathers deteriorate through time due to abrasion and exposure to sunlight and have to be renewed periodically. Molt, with its particular nutritional demands in terms of protein mobilization, is more flexible in timing than other annually repeated events (Jenni & Winkler, 2020; Lindstrom & Piersma, 1993; Newton, 2009, 2011).

Timing and pattern of molt in relation to migration varies, and flight feather replacement occurs either in breeding areas, wintering sites, or at stopover sites on migration. The annual schedule depends on species or individual migratory strategies (Carlisle et al., 2005; Ginn & Melville, 1983; Jenni & Winkler, 2020; Newton, 2009; Stresemann & Stresemann, 1966) and frequently differs also among different geographical populations of the same species (Hemborg et al., 2001; Remisiewicz, 2011).

In contrast to resident populations or birds that molt in winter quarters, migratory species that molt during late summer at high latitudes are less flexible regarding the timing of their molt and may undergo more rapid flight feather replacement before departure. But rapid molting results in openings in the wing and/or reduced feather quality, impeding flight efficiency, and increasing risk of predation (Butler, 2013; De La Hera et al., 2009; Hedenström & Sunada, 1999; Moller & Nielsen, 2018; Morrison et al., 2015; Rohwer et al., 2011; Serra et al., 2007; Swaddle & Winter, 1997; but see Bridge, 2011). Furthermore, molt‐related feather gaps in the wing or tail may increase energetic costs and simultaneously impair accumulation of energy reserves for migration. Molt speed and the sequence of feather renewal may affect the size and shape of molt‐related feather gaps with possible impacts on flight metabolism and performance during the molt process. Therefore, the timing of molt and extensive refueling rarely coincides (Barta et al., 2007). However, in some cases fuel deposition rate or body mass increase is little affected by molt (e.g., Holmgren et al., 1993).

Among almost all Western Palearctic passerines, the basic sequence of the complete primary molt is uniformly descendant, beginning with the innermost primary (P) P1, and the renewal of secondaries (S) begins ascendantly with S1 (Ginn & Melville, 1983; Jenni & Winkler, 2020). The few long‐distance migrants that molt twice per year may replace their primaries in an ascending, descending, or eccentric sequence. The latter pattern of eccentric primary molt does not start with P1, but somewhere in the center of the primaries or in the outer primaries (Jenni & Winkler, 2020). In some passerines, the starting point of primary molt may be shifted from P1 to a middle primary (divergent sequence: renewal proceeding simultaneously both inwards and outwards) or to the outermost P9 (ascendant sequence; Kulaszewicz & Jakubas, 2015; Neto & Gosler, 2006; Stein, 2012; Steiner, 1970; Thomas, 1977). However, divergent molt is still exceptional among Western Palearctic passerines, and the occurrence of this molt sequence might be correlated with accelerated molt speed (Kiat, 2017).

The need for rapid molt might be highest for species breeding in highly seasonal habitats, with limited time on the breeding grounds. Strongest seasonal changes in climate are found around the Siberian “cold pole,” with temperature ranges of more than 100°C. One of the latest arriving species breeding in Siberia is the Pallas´s Grasshopper Warbler *Locustella certhiola*, which stays only around two months on the breeding grounds (Bozo et al., 2019; Kennerley & Pearson, 2010; Sleptsov, 2018). Geolocator tracking revealed a very rapid migration in this species: A breeding bird from the Russian Far East covered a distance of almost 5,000 km in less than one month during autumn migration (Heim et al., 2020). These extreme time constraints might have led to the evolution of a peculiar molt strategy, but little information is available so far.

The Pallas's Grasshopper Warblers are known to undergo a complete prebreeding molt in which up to four primaries may be grown simultaneously during normal descendant molt in March–April, shortly before spring migration (Bub & Dorsch, 1995; Nisbet, 1967; Stresemann & Stresemann, 1972). On the other hand, Stresemann and Stresemann (1972) stated that adult Pallas's Grasshopper Warblers undergo a complete molt in autumn as well, and two individuals in active primary molt were found in November in Myanmar (Williamson, 1976). Kennerley and Pearson (2010) reported adult birds from Hong Kong in September which had three outer primaries presumably replaced before migration. Chernyshov (2017) reported an individual trapped on 25 August at Chany Lake in West Siberia with freshly molted primaries, tertials, rectrices, and body feathers. The westernmost breeding population may have the longest migration route of all populations and is supposed by Dementev and Gladkov (1954) to have a complete molt twice annually. In addition, Svensson (1992) mentioned also two birds from Turkestan in August undergoing an advanced active postbreeding molt and wondered whether adults “might undergo an extremely rapid complete (or nearly complete) molt in late summer‐early autumn” (Svensson, 1992). Furthermore, four adults with initiated but arrested wing feather molt were caught during autumn migration in Beidaihe, China (Norevik et al., 2020). These observations, though based on few individuals, suggest the occurrence of two molt seasons in the Pallas´s Grasshopper Warbler, a (partial) postbreeding molt, and a complete prebreeding molt.

Here, we ask the question whether the extreme time constraints resulting from a very short stay on the breeding grounds and rapid long‐distance migration have led to specific adaptations in the molt pattern of the Pallas´s Grasshopper Warbler. First, we analyze the extent and direction of primary molt and the frequency of observed molt patterns based on individuals caught at six sites situated along the migration route. Second, we analyze the correlation of postbreeding molt with body condition and fuel loads.

## 2 MATERIALS AND METHODS

### 2.1 Study sites

We compiled data on molt and body condition of Pallas´s Grasshopper Warblers from a total of six sites situated along its migration route (Table 1): from a breeding site at the northwestern limit of its distribution (Mirnoye, Central Siberia), at breeding and possible stopover sites in the Russian Far East (Muraviovka Park, Amur region) and Mongolia (Khurkh Bird Ringing Station) through stopover sites in South Korea (Heuksando Island) and Hong Kong (Mai Po) to a potential wintering site near Bangkok, Thailand (Figure 3). Birds were caught with mist‐nets without using playback.

**TABLE 1:** Extent of postbreeding molt in wing and tail feather tracts of the Pallas´s Grasshopper Warblers at six sites along the migration route (PP = primaries, SS = secondaries, TT = tertials, TF = tail feathers).

Study site	Central Siberia	Mongolia	Russian Far East	Korea	Hong Kong	Thailand
Sampling period	July–August	August–September	August–September	August	September	August–December
Sampling years	1990–2015	2018	2011–2017	2014–2016	2008–2018	1995–2014
PP						
*n* individuals	26	14	16	9	137	28
% Of ind having all/some molted	0%/100%	0%/100%	19%/100%	0%/100%		0%/75%
Mean number (range) of molted	3.3 (1–7)	6.0 (2–8)	7.3 (2–9)	7.1 (5–8)	8.0 (0–9)	5.0 (0–7)
SS						
*n* individuals	19		16	7		22
% Of ind having all/some molted	0%/0%		0%/62%	0%/57%		0%/14%
Mean number (range) of molted	0.0 (0)		0.8 (0–3)	0.7 (0–2)		
TT						
*n* individuals	19		16	7		24
% Of ind having all/some molted	32%/47%		56%/81%	100%/100%		63%/100%
Mean number (range) of molted	2.4 (2–3)		2.6 (0–3)	3.0 (3)		2.5 (1–3)
TF						
*n* individuals	19		15	7		
% Of ind having all/some molted	100%/100%		87%/100%	85%/85%		
Mean number (range) of molted	6.0 (6)		4.1 (0–6)	5.0 (0–6)		
Fat						
*n* individuals	6	14	13	9	183	24
Mean fat score	2.2	3.6	1.8	3.9	3.0	2.1
Muscle score						
*n* individuals		14	13			21
Mean muscle score		2.4	2.0			2.0
Mass						
*n* individuals	21	14	14	9	183	24
Mean body mass in g	15.4	15.3	14.7	16.0	13.7	16.0
Range in g	14.0–16.2	13.5–17.6	13.2–16.5	13.3–19.1		13.3–19.4

Note

Given is the mean number of molted feathers (range) and below the percentage of individuals having molted all/some of those feathers, if recorded.

### 2.2 Data collection and selection

We included only adult birds, as juveniles do not molt their flight feathers in autumn (Bub & Dorsch, 1995; Svensson, 1992). Aging in autumn was based on yellowish underpart color and dark spotting on the upper breast and lower throat in juveniles (Svensson, 1992). Body mass was taken to the nearest 0.1 g with an electronic scale. Visible subcutaneous fat load in the tracheal pit and on the abdomen was estimated on a nine‐point scale (0–8) according to Kaiser (1995). The thickness of the breast muscles was scored on a four‐digit scale (0–3) according to Bairlein (1995). Primaries (P1–P9) were numbered sequentially, descendantly, secondaries (S1–S6) and tertials (S7–S9) ascendantly.

Most detailed information on molt was collected at the study site in the Russian Far East. Here, the molt of remiges was recorded by attributing a score from zero (old feather) to five (completely grown new feather) to all flight feathers (Ginn & Melville, 1983). The total molt score (TMS) was determined for each bird by adding the molt scores of the individual nine long primaries and the six secondaries of the left wing, but excluding the very small outermost primary (P10), to give a maximum TMS of 45 for primaries and 30 for secondaries. When molting birds (i.e., birds in active wing feather molt) were recaptured, molt score was again calculated and used to estimate molt progress per day, calculated as the difference in TMS divided by the number of days between capture and recapture. Due to a lack of data on feather mass in this species, it was assumed that the primary molt score was linearly related to time (but see Newton, 2009; Underhill & Zucchini, 1988).

### 2.3 Statistical analysis

We compared the percentage of individuals showing postbreeding primary molt and the number of molted primaries as well as the direction of molt. In a second step, we modeled the number of molted primaries as an index for molt progress using linear mixed‐effect models in R package lme4 (Bates et al., 2014). *Julian day* and *latitude* were fitted as fixed independent effects. *Study site* was included as random effect. We built two sets of candidate models, one including all birds trapped during the autumn migration period (15 July–25 September, based on the bird tracked by Heim et al., 2020) and one including only individuals trapped at stopover sites (excluding the likely wintering site in Thailand). We graphically examined if model assumptions were met using residual plots and evaluated model fit with the conditional *R* ^2^ and marginal *R* ^2^ (Nakagawa & Schielzeth, 2013). Fixed effects were tested with a likelihood ratio test.

To test whether flight feather molt is connected to body condition, we examined molting and nonmolting birds caught between 27 July and 14 September (a time when both molting and nonmolting birds co‐occur) at the study site in the Russian Far East. The relationship between molt and body mass was estimated by linear regression using log‐body mass. The correlation of molt (yes/no) with fat and muscle score was estimated using a Wilcoxon–Mann–Whitney test as both fat and muscle score were measured at ordinal scales and were not normally distributed.

Data analyses were conducted using the software R (R Core Team, 2017). The additional packages *xlsx* (Dragulescu & Arendt, 2018), *reshape2* (Wickham, 2007), and *ggplot2* (Wickham, 2016) were needed for data import, aggregating data, and creating graphs, respectively.

## 3 RESULTS

### 3.1 Postbreeding molt patterns

In Central Siberia, nearly two thirds of adult individuals caught in July and August (*n* = 26) had recently commenced their primary molt with the starting point at the center of the feather tract; therefore, the molt patterns of these 16 birds (62%) were unidentifiable (Table 1). Feather renewal of 8 individuals (31%) followed a divergent sequence, while one bird probably showed a descendant molt pattern. The approximate molting trajectory was similar to that of birds from the Russian Far East, although molt started earlier in Central Siberia (Figure 2b).

In the Russian Far East, 65 out of 190 adult Pallas's Grasshopper Warblers caught between May and September showed signs of postbreeding molt (Table 2, Figure S1). Detailed descriptions of flight feather renewal of adult birds in the Russian Far East were available only for birds caught in 2017 (*n* = 16, Table 1). The first adult bird molting its primaries (P3–P6) was caught on 27 July 2017 (TMS not recorded). The earliest bird to have nearly completed primary molt (TMS 43) was caught on 16 August, the latest bird with completed molt on 27 August.

**TABLE 2:** Number of Pallas's Grasshopper Warblers molting and not molting flight feathers caught between 2011 and 2017 in the Russian Far East summarized by ten‐day periods (decade)

Decade	Molting	Not molting	% Molting
1: 28 May–6 June	0 (0)	23 (27)	0 (0)
2: 7 June–16 June	0 (0)	16 (27)	0 (0)
3: 17 June–26 June	0 (0)	19 (26)	0 (0)
4: 27 June–6 July	0 (0)	14 (19)	0 (0)
5: 7 July–16 July	0 (0)	8 (14)	0 (0)
6: 17 July–26 July	0 (0)	9 (11)	0 (0)
7: 27 July–5 August	13 (18)	14 (23)	48 (44)
8: 6 August–15 August	15 (19)	5 (8)	75 (70)
9: 16 August–25 August	24 (32)	7 (10)	77 (76)
10: 26 August–4 September	9 (12)	9 (14)	50 (46)
11: 5 September–14 September	3 (4)	1 (3)	75 (57)
12: 15 September–24 September	1 (1)	0 (0)	100 (100)
Sum	65 (86)	125 (182)	34 (32)

Note

Numbers without brackets represent first handlings of birds, and numbers within brackets represent first traps and retraps within and between years. Total *N*_without retraps_ = 190; total *N*_with retraps_ = 268.

**FIGURE 1:**
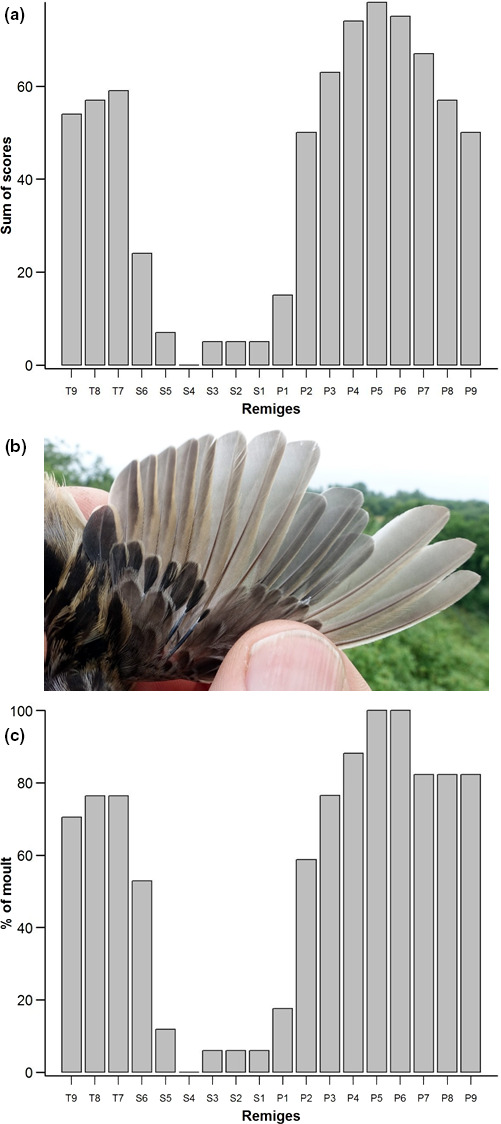
(a) Postbreeding molt sequence of primaries (P), secondaries (S), and tertials (T) in the Pallas's Grasshopper Warblers in the Russian Far East (2017, *n* = 16). (b) *The* Pallas's Grasshopper Warbler *Locustella certhiola* in the postbreeding molt at Muraviovka Park/Russian Far East, 3 August 2017: primaries molted divergently (photo credit: H.‐J. Eilts). (c) Extent of postbreeding molt in the Pallas's Grasshopper Warblers (*n* = 16). % of molt = percentage of birds that replaced each remex

The individual scores of each remex (wing feather) of molting adults in the Russian Far East were summed to determine the general sequence of molt in this population (Figure 1a). The postbreeding molt tended to be initiated in P5, nearly simultaneously followed by P6 and P4 (Figure 1b,c). Ten individuals (63%) underwent divergent molt. The median number of replaced primaries within this group was 7.3, with a range from four to eight (seven individuals). Three further birds (19%) were probably also replacing their wing feathers in a divergent way (e.g., P4 > P5 > P3 > P6 > P2 > P7 > P1 > P8 > P9), but since the inner primaries were nearly fully grown, the precise molt sequence in these individuals was unknown. One adult showed an eccentrically descendant molt pattern, that is, starting from the middle primaries and then outwards, while one individual had just started molt with P5 and P6 emerging from the sheath. Only 3 out of 16 birds had replaced all primaries (Table 1), that is, were completing primary molt except for the two outermost primaries (still growing, each with score 4). P5 and P6 were invariably molted, whereas 81% of the birds retained an unmolted P1 and 38% retained P2, too (Figure 1c). The retained, unmolted primaries were generally located in the inner wing, regardless of whether the molt pattern was divergent or eccentrically descendant.

We found that 56% of the birds molted their tertials completely, most often following the sequence T7–T8–T9 (Table 1). Single secondaries were shed quite unsystematically and without any clear connection with the sequence and extent of primary molt. In 53% of the birds S6 was shed, descendantly followed by S5 (Figure 1c). Only three adults had completely replaced S1–S3. Tail feather molt occurred in 87% (Table 1). Only one individual renewed some primary coverts, which did not molt at the same time or in the same sequence as their corresponding primaries. Most individuals had nearly completed their body feather molt except for the dorsal tract, and a few birds underwent a complete molt of their whole body plumage.

**FIGURE 2:**
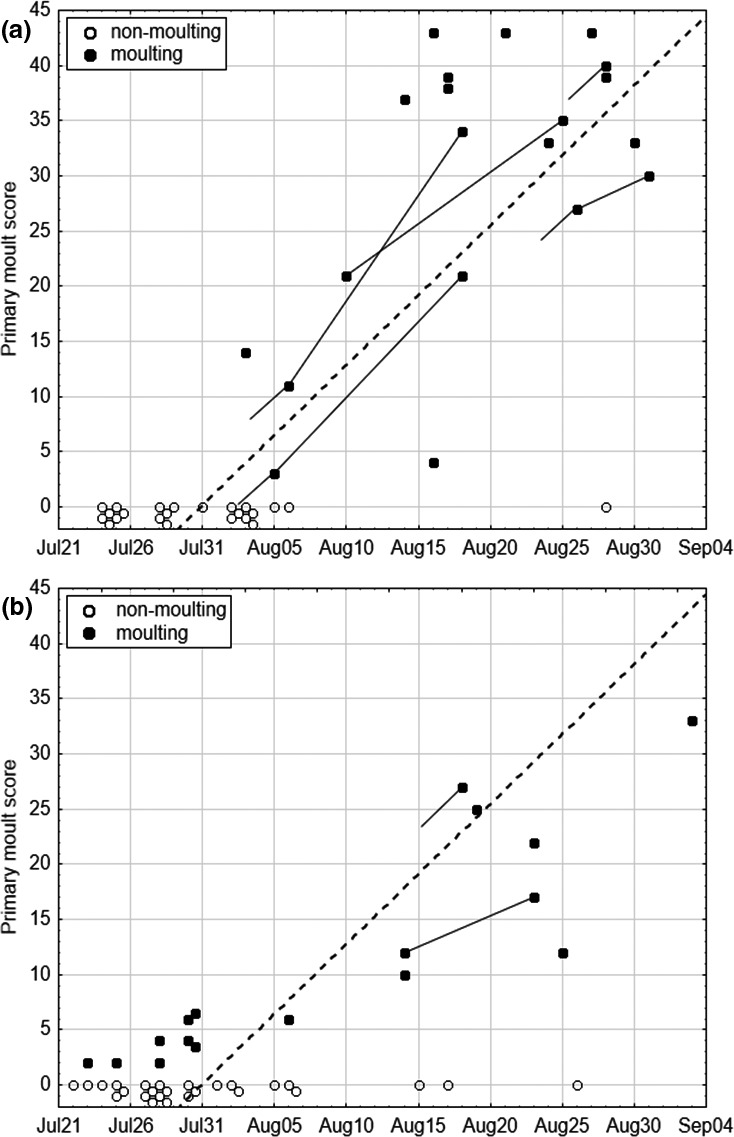
Individual primary molt scores of the Pallas´s Grasshopper Warblers studied in detail in (a) the Russian Far East and (b) Central Siberia. Lines connect observations of recaptured individuals, and lines starting without point correspond to retraps following a catch before molt started. Dashed line approximates the average individual molting rate based on retraps

Based on four recaptures of actively molting adults in the Russian Far East, the weighted arithmetic mean rate of primary molt was 1.26 molt scores per day (range 0.50–1.92), assuming continuous (uninterrupted) molt (Figure 2a). When the secondaries were included, the weighted arithmetic mean rate was 1.98 molt scores per day (range 0.50–2.83). Hence, the Pallas's Grasshopper Warblers would need on average 36 days (range 23–90) to complete their postbreeding primary molt, or 38 days (range 26–150) if the secondaries were included. We estimated the start of molt for each of the four birds individually by using their individual molt speed. On average, postbreeding molt started on 21 July, if only primaries are considered, or on 15 July, if primaries and secondaries are considered.

In Mongolia, all birds (*n* = 14) showed the normal descendant molt sequence of the primaries during August and September. 11 individuals (79%) had interrupted their molt with a median number of six replaced primaries (range four to eight), while three birds were still molting with two growing feathers in each case (Table 1).

In contrast to the above, 50% of the Pallas's Grasshopper Warblers examined in Korea during August (*n* = 9) had already replaced all their primaries, except for the innermost (on average two) remaining old feathers (Table 1). In addition to replacing all tertials, three out of nine individuals had also molted S6. However, the proportion of birds that might have molted primaries in an eccentrically descendant or divergent pattern prior to their arrival in Korea is unknown.

The molt sequence and extent among birds trapped during autumn passage through Hong Kong, mostly in September (*n* = 137, predominantly *L. c. certhiola/minor* with a few *L. c. rubescens*), was only slightly different. Only 4% of the individuals had molted fewer than five primaries (Table 1). Typically, inner primaries, secondaries, and all primary coverts were unmolted. Because none were in active molt, the proportion of individuals that had undergone eccentrically descendant and potential divergent feather renewal prior to arrival in Hong Kong could not be determined.

Further south, in Thailand, 75% of the birds (*n* = 28, presumably mainly *L. c. rubescens*) showed signs of having previously undergone a partial postbreeding primary molt (Table 1). Most replaced feathers were observed in the center of the feather tract, with unknown sequence. Worth mentioning are seven individuals with an “old,” unmolted primary tract, carried over from their preceding spring molt. 86% of the birds in Thailand had not molted any secondaries, while 63% had renewed all three tertials and in one bird T9 was growing.

Our models revealed that the number of molted primaries increased significantly with the progress of the autumn season, and possibly with decreasing latitude (Table 3). However, no effect of latitude was found when data from the wintering site were included.

**TABLE 3:** Effects of Julian day and latitude on the number of molted primaries using linear mixed‐effects models, based on 127 Pallas´s Grasshopper Warblers

Models	Julian day	Latitude	*R* ^2^
1 (all six sites)	*χ* ^2^ = 26.07 *p* < .001	*χ* ^2^ = 2.15 *p* = .14	*R*_cond_ = .68 *R*_marg_ = .15
2 (excluding wintering site)	*χ* ^2^ = 22.00 *p* < .001	*χ* ^2^ = 3.95 *p* = .05	*R*_cond_ = .72 *R*_marg_ = .58

Note

Significant effects are highlighted with gray background.

### 3.2 Fuel loads and effects of molt on body condition

Lowest fat scores were found at the breeding site in Central Siberia, at the breeding and stopover site in the Russian Far East and at the potential wintering site in Thailand (Table 1). Muscle scores were available from three sites, with the highest mean scores found at the breeding site in Mongolia and moderate muscle scores found in molting birds in the Russian Far East and in birds in Thailand. Mean body masses found during migration in Korea and in Thailand were highest, while lowest body masses were found during stopover in Hong Kong. In the Russian Far East, body mass of molting (mean 14.3 g ± standard deviation 1.0 g, range 12.3–17.3 g, *n* = 62) and nonmolting birds (mean 14.5 g ± standard deviation 1.2 g, range 12.1–18.5 g, *n* = 36) did not differ significantly in the time window in which both were observed (*b* = 0.02, *t* = 1.3, *p* = .19). Nor did fat scores (median score: 2) of molting and nonmolting birds differ significantly (*W* = 1,210, *p* = .57), though muscle scores (median score: 2) of nonmolters were significantly higher (*W* = 1,356.5, *p* = .01; Figure S2 and Tables S1–S3).

## 4 DISCUSSION

### 4.1 Postbreeding molt

This is the first study providing direct evidence for a partial postbreeding molt of the Pallas's Grasshopper Warblers. With 36 days, the duration of the primary molt (outermost primary excluded) of birds caught in the Russian Far East took place in significantly less time than the 50–60 days estimated by Nisbet (1967) for prebreeding molters in spring. The accelerated partial postbreeding molt might be caused by the late time of nesting, the long migration distance, and associated time constraints due to decreasing food availability in seasonal habitats on their temperate breeding grounds (Ginn & Melville, 1983; Newton, 2009). The shorter molt duration might also stem from differences in the extent of molt, as many individuals do not finish their primary molt on the breeding grounds, or from the low sample size. We suggest that besides a premigratory molt of local breeders prior to autumn migration, a percentage of the trapped individuals with signs of advanced primary molt exhibited a temporal overlap between commenced migratory movement and flight feather replacement or a suspended molt migration (Tonra & Reudink, 2018). A possible confirmation of these two potential molt strategies is provided by the comparison of primary molt dynamics of the Pallas´s Grasshopper Warblers in Central Siberia and the Russian Far East, each site using a constant‐effort schedule. Only few birds of the Central Siberian population molt on the place of breeding (Figure 2b), most of them depart around mid‐August before or at the very beginning of molt. In the Russian Far East, many birds at terminal stages of primary renewal appear in the second half of August (Figure 2a). They are unlikely to have started molt at this site if their molt follows the progress of the four earlier reported retrapped individuals. Thus, these birds with terminating molt might belong to northern populations, such as from Central Siberia. Molt migration seems to be relatively common in Neotropical migrants (Leu & Thompson, 2002) and has been linked to aridity of the breeding grounds (Pageau et al., 2020).

Based on one geolocator‐tracked individual that left its breeding site on 2 September (and arrived in its wintering area in Thailand already on 25 September; Heim et al., 2020), adult birds of the population in the Russian Far East likely depart in late August or the beginning of September. Only two of the 16 birds examined in detail in the Russian Far East were judged to have interrupted their wing molt, arresting after growing P4–P9 and P3–P9, respectively. Thus, it cannot be ruled out that most individuals molt continuously during active migration, maybe encouraged by continuous suitable environmental conditions en route to their South‐East Asian wintering areas (Heim et al., 2020; Yong et al., 2015). Similar observations of molt bridging autumn migration have been made in other songbirds (Elkins & Etheridge, 1977; Herremans, 1990; Kiat et al., 2018; Nisbet & Medway, 1971; Schaub & Jenni, 2000b).

Based on our data, the Pallas's Grasshopper Warbler is the first eastern Palearctic passerine for which a divergent primary molt pattern is demonstrated (Figure 1b). Regular descendant sequence has been documented in this species too, although to a much smaller extent than divergent molt. Similar observations were made in Savi´s Warblers *Locustella luscinoides* in Europe (Thomas, 1977). The distribution curve of molted remiges (Figure 1c) was also similar to that of Savi's Warblers showing divergent wing molt (Thomas, 1977). According to Kiat (2017), the benefit of a divergent molt strategy is that the wing gap comes about shortly after the start of the primary molt and is later split into two smaller gaps rather than one larger wing gap during the normal descendant molt sequence. This might allow divergent molters a higher molt speed and lower aerodynamic costs than in descendant molters (Kiat, 2017, 2018, but see Bridge, 2011).

Together with the tertials, S6 was usually molted (“abridged” molt, Norman, 1991) in the Pallas´s Grasshopper Warblers in the Russian Far East, as shown for adults of other long‐distance migrants with a partial postbreeding molt (Jenni & Winkler, 2020; Svensson, 1992), followed by S5. This might indicate that secondary molt proceeds convergently, a frequent exception in partial secondary molt (Jenni & Winkler, 2020; Neto & Gosler, 2006). Retaining the central secondaries, usually the least abraded of remaining wing feathers (Norman, 1986), shortens molt duration and presumably allows resources to be diverted preferentially to primary feather synthesis. The centers of primary and tertial molt, arranged around P5/6 and T8, are thought to have an adaptive function (Mester & Prünte, 1982). In the folded wing of a primarily reed foraging species, such as the Pallas´s Grasshopper Warbler, they might serve as protective shields against forced abrasion and sun exposure for the outermost pointed primaries and the central secondaries, respectively. Simultaneously, P5 and its adjacent feathers are exposed to intense wear, quasi representatively for the important outer wing feathers regarding aerodynamic and flight efficiency (Lockwood et al., 1998).

**FIGURE 3:**
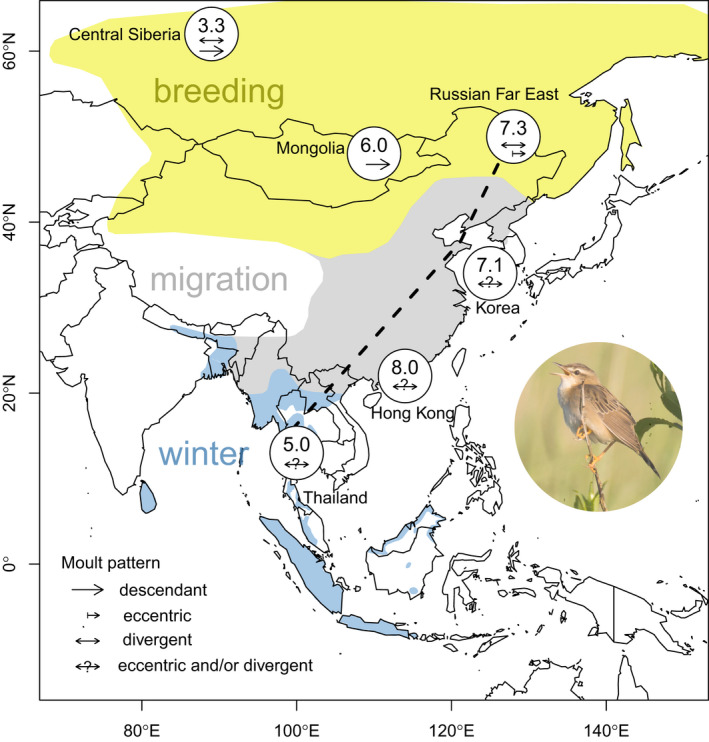
Mean number of molted primaries and molt pattern(s) of the Pallas´s Grasshopper Warblers at six sites along the East Asian Flyway. The dashed line indicates the migration route of a geolocator‐tracked individual (Heim et al., 2020). Distribution based on BirdLife International (2020). Photo credit: Arend Heim/Amur Bird Project

Interestingly, the unmolted primaries of the majority of adult Pallas's Grasshopper Warblers in Central Siberia and the Russian Far East appeared in pristine condition. Rather than “exceptional strength” (cf. Svensson, 1992), this might more likely relate to the very recent prebreeding wing molt pre‐dating the onset of spring migration in mid‐May. It further highlights the open question of the reasons for the variation in postbreeding primary molt patterns of this species. The above‐mentioned group of adult nonmolters could belong to a population from northern parts of the breeding range (*L. c. rubescens*) which might completely postpone their molt until arrival in wintering areas (Newton, 2009) or which have not yet started their flight feather renewal. However, based on our data from Central Siberia, at least some of the northernmost breeders start to molt on the breeding grounds. Little is known about population‐specific nonbreeding areas, except for the tracked individual connecting the Russian Far East and Thailand (Figure 3). Our molt data support the finding that some birds at the Bangkok ringing site in Thailand could belong to the breeding population from the Russian Far East.

Our results indicate that intraspecific variation in molt sequence can neither be attributed to specific regions of the species’ vast breeding range, nor be attributed to a subspecies. Based on our data from the Russian Far East, plasticity in postbreeding primary molt sequence cannot be easily related to season either, contrary to observations in another species with eccentric molt (Neto & Gosler, 2006).

It should be added that in spring, apart from four actively molting birds with irregularities in the molt sequence that cannot be allocated, 16 individuals caught during February–May 1995–2014 in Thailand followed the basic molt sequence of primaries (P. Round, unpublished data). These records indicate that partial postbreeding flight feather renewal in adult Pallas´s Grasshopper Warblers in general represents a separate molt that is arrested both within primaries and secondaries, followed by a second complete prebreeding molt in the nonbreeding area, as assumed for other *Locustella* species as well (Jenni & Winkler, 2020; Pearson & Backhurst, 1983; Svensson, 1992).

Tail feathers in the Pallas's Grasshopper Warbler are dropped simultaneously (Nisbet, 1967), as in other *Acrocephalus* and *Locustella* warblers (Kennerley & Pearson, 2010; Round & Rumsey, 2003). The specific tail and primary molt in the Pallas's Grasshopper Warbler might be supported by its feeding mainly in reeds and dense scrub, without having to cross wide gaps and thus saving energetic costs connected to movement with temporary wing and tail feather deficits. This behavior is shared by other *Locustella* species (Kennerley & Pearson, 2010) and might explain why the occurrence of divergent molt seems to be more widespread in this group.

### 4.2 Molt migration and body condition

New production of flight and body feathers requires protein synthesis, diverting energy (Murphy & King, 1992), which cannot be allocated to fuel reserves in preparation for migration. Therefore, average fuel deposition rates and fat scores should be lower in molting birds (Merila, 1997; Remisiewicz et al., 2018; Rubolini et al., 2002; Schaub & Jenni, 2000a). Contrarily, we found constantly moderate body mass levels and low fat scores at our study site in the Russian Far East, with no significant differences in fat scores between molting and nonmolting birds, nor any remarkable increase later in the season in either group. Although comparatively more nonmolting birds had a maximum muscle score, flight muscle scores on average remained moderate throughout the season in both groups, indicating a low protein catabolism within molting birds, presumably because the demand for protein was easily met by dietary intake (Jenni‐Eiermann & Jenni, 1996; Murphy & King, 1992; Podlaszczuk et al., 2017). The Pallas's Grasshopper Warblers are migrating largely over land without facing any major ecological barriers (Heim et al., 2020; Kennerley & Pearson, 2010). According to optimality models (Alerstam, 2011; Alerstam & Lindström, 1990), this would make longer nonstop flights redundant and would instead allow a nocturnal flight strategy with low fuel loads (Figure 3) and daily refueling in suitable habitats. Neither muscle hypertrophy in preparation for migration (Lindström & Piersma, 1993; Lundgren & Kiessling, 1985) nor the need to catabolize flight muscle protein is evident. A similar migration strategy seems to be a frequently used pattern in nocturnal passerine migrants flying across continental Europe where widespread fueling opportunities exist (Bairlein, 1995; Delingat et al., 2006; Ozarowska, 2015; Schaub & Jenni, 2000a; Stepniewska et al., 2018). Recent studies found huge variation in fuel loads among migratory warblers in East Asia, with comparatively low fuel loads in the Pallas´s Grasshopper Warblers (Bozo et al., 2020; Sander et al., 2017, 2019). Further support for an autumn migration with low fuel loads is found by individuals examined at the stopover site in Hong Kong. In contrast, the capture of heavy birds in Thailand possibly indicates that substantial migratory refueling might occur at this stage for those individuals migrating further south, for example, for crossing the gulf of Thailand on to Sundaland. On the Malay Peninsula, adult (presumably nonmolting) birds passing through in September were slightly heavier than overwintering, nonmolting individuals during October–mid‐March (Nisbet, 1967). Different origins/subspecies could also play a role, as northern/western populations are somewhat larger than southern/eastern populations (Kennerley & Pearson, 2010).

## 5 CONCLUSIONS

This is the first study combining data on molt from six different sites for a songbird species migrating along the East Asian Flyway. By investigating molt patterns of the Pallas's Grasshopper Warbler, we have gained valuable insights into a central aspect of its migration strategy that has not been previously documented. Our results demonstrate that this species undertakes a rapid, partial postbreeding molt of flight feathers. Particularly interesting is the variation of primary molt patterns within and between different populations/subspecies and geographical regions, as has been revealed in this study. To our knowledge, this is the first time that the rare divergent molt pattern has been witnessed among Palearctic passerines in East Asia. At the same time, a considerable proportion of birds did not show signs of primary renewal. Plasticity of this kind could be highly advantageous to the Pallas's Grasshopper Warblers in regulating the energetic costs of overlapping late breeding commitment and timing of molt and migration. Further detailed studies may shed light on the impacts on the individual molt strategies within a population and among individuals in response to variation in climate or habitats (Newton, 2011).

## CONFLICT OF INTEREST

None declared.

## AUTHOR CONTRIBUTIONS

**Hans‐Jürgen Eilts:** Conceptualization (lead); Data curation (equal); Formal analysis (lead); Investigation (equal); Writing‐original draft (lead); Writing‐review & editing (lead). **Nele Feuerbach:** Data curation (equal); Formal analysis (equal); Writing‐review & editing (supporting). **Philip D. Round:** Investigation (equal); Writing‐original draft (equal); Writing‐review & editing (equal). **Oleg Bourski:** Data curation (equal); Formal analysis (equal); Investigation (equal); Writing‐review & editing (equal). **John Allcock:** Investigation (equal); Writing‐review & editing (supporting). **Paul J. Leader:** Investigation (equal); Writing‐review & editing (supporting). **Davaasuren Batmunkh:** Investigation (equal); Writing‐review & editing (supporting). **Tuvshinjargal Erdenechimeg:** Investigation (equal); Writing‐review & editing (supporting). **Jong‐Gil Park:** Investigation (equal); Writing‐review & editing (supporting). **Wieland Heim:** Conceptualization (lead); Data curation (equal); Formal analysis (equal); Investigation (equal); Project administration (lead); Writing‐original draft (lead); Writing‐review & editing (lead).

## Supporting information

Supplementary Material

## Data Availability

Data used for the models on molt progress are available on Dryad (https://doi.org/10.5061/dryad.vq83bk3rc). Further data are available upon request from the authors.

## References

[ece37098-bib-0001] Alerstam, T. (2011). Optimal bird migration revisited. Journal of Ornithology, 152(Suppl.), 1–46.

[ece37098-bib-0002] Alerstam, T. , & Lindström, A. (1990). Optimal bird migration: The relative importance of time, energy, and safety In Gwinner E. (Ed.), Bird migration: Physiology and ecophysiology (pp. 331–351). Springer Verlag.

[ece37098-bib-0003] Bairlein, F. (1995). European‐African songbird migration network manual of field methods. Vogelwarte Helgoland.

[ece37098-bib-0004] Barta, Z. , McNamara, J. M. , Houston, A. I. , Weber, T. P. , Hedenström, A. , & Fero, O. (2007). Optimal moult strategies in migratory birds. Philosophical Transactions of the Royal Society B: Biological Sciences, 363, 211–229. 10.1098/rstb.2007.2136 PMC2606747 17681914

[ece37098-bib-0005] Bates, D. , Maechler, M. , Bolker, B. , & Walker, S. (2014). lme4: Linear mixed‐effects models using Eigen and S4. R Package Version, 1(7), 1–23.

[ece37098-bib-0006] BirdLife International (2020). Species factsheet: *Locustella certhiola*. Retrieved from http://www.birdlife.org

[ece37098-bib-0007] Bozo, L. , Csörgo, T. , & Anisimov, Y. (2020). Estimation of flight range of migrant leaf‐warblers at Lake Baikal. Ardeola, 67(1), 101–111.

[ece37098-bib-0008] Bozo, L. , Heim, W. , Trense, D. , Fetting, P. , Eilts, H.‐J. , Wobker, J. , & Csörgo, T. (2019). Migration of Pallas’s Grasshopper Warbler and Lanceolated Warbler at a Far Eastern Russian stopover site. Ornithological Science, 18(2), 177–181.

[ece37098-bib-0009] Bridge, E. S. (2011). Mind the gaps: What’s missing in our understanding of feather moult. The Condor, 113, 1–4.

[ece37098-bib-0010] Bub, H. , & Dorsch, H. (1995). Kennzeichen und Mauser europäischer Singvögel 4: Cistensänger, Seidensänger u.a. Die Neue Brehm‐Bücherei Band 580. Westarp Wissenschaften.

[ece37098-bib-0011] Butler, L. K. (2013). The grass is always greener: Do monsoon rains matter for molt of the vermilion Flycatcher (*Pyrocephalus rubinus*)? The Auk, 130, 297–307.

[ece37098-bib-0012] Carlisle, J. D. , Kaltenecker, G. S. , & Swanson, D. L. (2005). Molt strategies and age differences in migration timing among autumn landbird migrants in southwestern Idaho. The Auk, 122, 1070–1085.

[ece37098-bib-0013] Chernyshov, V. M. (2017). Cases of catching Yellow‐headed Wagtail *Motacilla lutea* and Pallas’s Grasshopper Warbler *Locustella certhiola* at Chany lake (South‐western Siberia). Russian Ornithological Journal, 26, 4020–4021 (in Russian).

[ece37098-bib-0014] De La Hera, J. , Perez‐Tris, J. , & Telleria, J. L. (2009). Migratory behaviour affects the trade‐off between feather growth rate and feather quality in a passerine bird. Biological Journal of the Linnean Society, 97, 98–105.

[ece37098-bib-0015] Delingat, J. , Dierschke, V. , Schmaljohann, H. , Mendel, B. , & Bairlein, F. (2006). Daily stopovers as optimal migration strategy in a long‐distance migrant passerine: The Northern Wheatear *Oenanthe oenanthe* . Ardea, 94, 593–605.

[ece37098-bib-0016] Dementev, G. P. , & Gladkov, N. A. (1954). Birds of the Soviet Union, Vol. VI Sov. Nauka.

[ece37098-bib-0017] Dragulescu, A. A. , & Arendt, C. (2018). xlsx: Read, write, format Excel 2007 and Excel 97/2000/XP/2003 files. R package version 0.6.1. Retrieved from https://CRAN.R‐project.org/package=xlsx

[ece37098-bib-0018] Elkins, N. , & Etheridge, B. (1977). Further studies of wintering Crag Martins. Ringing & Migration, 1, 158–165.

[ece37098-bib-0019] Ginn, H. B. , & Melville, H. D. (Eds.). (1983). Moult in birds In BTO Guide, 19, pp. 1–112. British Trust for Ornithology.

[ece37098-bib-0020] Hedenström, A. , & Sunada, S. (1999). On the aerodynamics of moult gaps in birds. Journal of Experimental Biology, 202, 67–76. 10.1242/jeb.202.1.67 9841896

[ece37098-bib-0021] Heim, W. , Heim, R. J. , Beermann, I. , Burkovskiy, O. A. , Gerasimov, Y. , Ktitorov, P. , Ozaki, K. , Panov, I. , Sander, M. M. , Sjöberg, S. , Smirenski, S. M. , Thomas, A. , Tottrup, A. P. , Tiunov, I. M. , Willemoes, M. , Hölzel, N. , Thorup, K. , & Kamp, J. (2020). Using geolocator tracking data and ringing archives to validate citizen‐science based seasonal predictions of songbird distribution in a data‐poor region. Global Ecology and Conservation, 24, e01215.

[ece37098-bib-0022] Hemborg, C. , Sanzz, J. J. , & Lundberg, A. (2001). Effects of latitude on the trade‐off between reproduction and moult: A long‐term study with Pied Flycatcher. Oecologia, 129, 206–212. 28547598 10.1007/s004420100710

[ece37098-bib-0023] Herremans, M. (1990). Body‐moult and migration overlap in reed warblers (*Acrocephalus scirpaceus*) trapped during nocturnal migration. Gerfaut, 80, 149–158.

[ece37098-bib-0024] Holmgren, N. , Ellegren, H. , & Pettersson, J. (1993). Stopover length, body mass and fuel deposition rate in autumn migrating adult Dunlins *Calidris alpina*: Evaluating the effects of moulting status and age. Ardea, 81, 9.

[ece37098-bib-0025] Jenni, L. , & Winkler, R. (2020). Moult and ageing of European passerines (2nd ed.). Helm.

[ece37098-bib-0026] Jenni‐Eiermann, S. , & Jenni, L. (1996). Metabolic differences between the postbreeding, moulting and migratory periods in feeding and fasting passerine birds. Functional Ecology, 10, 62–72.

[ece37098-bib-0027] Kaiser, A. (1995). A new multi‐category classification of subcutaneous fat deposits of songbirds. Journal of Field Ornithology, 64, 246–255.

[ece37098-bib-0028] Kennerley, P. , & Pearson, D. (2010). Reed and bush warblers. Christopher Helm.

[ece37098-bib-0029] Kiat, Y. (2017). Divergent primary moult‐ A rare moult sequence among Western Palaearctic passerines. PLoS One, 12, e0187282. 29088288 10.1371/journal.pone.0187282 PMC5663452

[ece37098-bib-0030] Kiat, Y. (2018). Divergent rectrix moult: The implications and conditions of moult sequence. Journal of Avian Biology, 49, jav‐01609.

[ece37098-bib-0031] Kiat, Y. , Izhaki, I. , & Sapir, N. (2018). The effects of long‐distance migration on the evolution of moult strategies in Western‐Palearctic passerines. Biological Reviews, 94, 700–720. 30334341 10.1111/brv.12474

[ece37098-bib-0032] Kulaszewicz, J. , & Jakubas, D. (2015). Factors affecting post‐breeding moult in the Savi’s Warbler *Locustella luscinoides* . Ardea, 103, 61–68.

[ece37098-bib-0033] Leu, M. , & Thompson, C. W. (2002). The potential of migratory stopover sites as flight feather molt staging areas: A review for neotropical migrants. Biological Conservation, 106, 45–56.

[ece37098-bib-0034] Lindström, A. , & Piersma, T. (1993). Mass changes in migrating birds: The evidence for fat and protein storage re‐examined. Ibis, 135, 70–78.

[ece37098-bib-0035] Lockwood, R. , Swaddle, J. P. , & Rayner, J. M. (1998). Avian wingtip shape indices and morphological adaptations on migration. Journal of Avian Biology, 29, 273–292.

[ece37098-bib-0036] Lundgren, B. O. , & Kiessling, K.‐H. (1985). Seasonal variation in catabolic enzyme activities in breast muscle of some migratory birds. Oecologia, 66, 468–471. 28310784 10.1007/BF00379335

[ece37098-bib-0038] Merila, J. (1997). Fat reserves and moult‐migration overlap in goldcrests *Regulus regulus* – A trade‐off? Annales Zoologici Fennici, 34, 229–234.

[ece37098-bib-0039] Mester, H. , & Prünte, W. (1982). Die “sektoriale” postjuvenile Handschwingenmauser der Carduelinen in Südeuropa. Journal Für Ornithologie, 123, 381–399.

[ece37098-bib-0040] Møller, A. P. , & Nielsen, J. T. (2018). The trade‐off between rapid feather growth and impaired feather quality increases risk of predation. Journal of Ornithology, 159, 165–171.

[ece37098-bib-0041] Morrison, C. A. , Baille, S. R. , Clark, J. A. , Johnston, A. , Leech, D. , & Robinson, R. R. (2015). Flexibility in the timing of post‐breeding moult in passerines in the UK. Ibis, 157, 340–350.

[ece37098-bib-0042] Murphy, M. E. , & King, J. R. (1992). Energy and nutrient use during moult by White‐crowned Sparrows *Zonotrichia leucophrys gambelii* . Ornis Scandinavica, 23, 304–313.

[ece37098-bib-0043] Nakagawa, S. , & Schielzeth, H. (2013). A general and simple method for obtaining R2 from generalized linear mixed‐effects models. Methods in Ecology and Evolution, 4(2), 133–142.

[ece37098-bib-0044] Neto, J. M. , & Gosler, A. G. (2006). Post‐juvenile and post‐breeding moult of Savi’s Warbler. Ibis, 148, 39–49.

[ece37098-bib-0045] Newton, I. (2009). Moult and plumage. Ringing & Migration, 24, 220–226.

[ece37098-bib-0046] Newton, I. (2011). Migration within the annual cycle: Species, sex and age differences. Journal of Ornithology, 152(Suppl.), 169–185.

[ece37098-bib-0047] Nisbet, J. C. T. (1967). Migration and moult in Pallas’s Grasshopper Warbler. Bird Study, 14, 96–103.

[ece37098-bib-0048] Nisbet, J. C. T. , & Medway, L. (1971). Dispersion, population ecology and migration of Eastern Great Reed Warblers *Acrocephalus orientalis* wintering in Malaysia. Ibis, 114, 451–494.

[ece37098-bib-0049] Norevik, G. , Hellström, M. , Liu, D. , & Petersson, B. (2020). Ageing & sexing of migratory East Asian passerines. Avium Förlag AB.

[ece37098-bib-0050] Norman, S. C. (1986). Feather abrasion in resident and migrant birds. South Cleveland Ringing Group Report, 8, 17–24.

[ece37098-bib-0051] Norman, S. C. (1991). Suspended split‐moult systems – An alternative explanation for some species of Palearctic migrants. Ringing & Migration, 12, 135–138.

[ece37098-bib-0052] Ozarowska, A. (2015). Contrasting fattening strategies in related migratory species: The blackcap, garden warbler, common whitethroat and lesser whitethroat. Annales Zoologici Fennici, 52, 115–212.

[ece37098-bib-0053] Pageau, C. , Tonra, C. M. , Shaikh, M. , Flood, N. J. , & Reudink, M. W. (2020). Evolution of moult‐migration is directly linked to aridity of the breeding grounds in North American passerines. Biology Letters, 16, 20200155. 32516565 10.1098/rsbl.2020.0155 PMC7336858

[ece37098-bib-0055] Pearson, D. J. , & Backhurst, G. C. (1983). Moult in the River Warbler *Locustella fluviatalis* . Ringing & Migration, 4, 227–230.

[ece37098-bib-0056] Podlaszczuk, P. , Wlodarczyk, R. , Janiszewski, T. , Kaczmarek, K. , & Minias, P. (2017). When moult overlaps migration: Moult‐related changes in plasma biochemistry of migrating common snipe. PeerJ, 5, e3057. 28286713 10.7717/peerj.3057 PMC5344014

[ece37098-bib-0057] R Core Team (2017). R: A language and environment for statistical computing. R Foundation for Statistical Computing Retrieved from https://www.R‐project.org/

[ece37098-bib-0058] Remisiewicz, M. (2011). The flexibility of primary moult in relation to migration in Palaearctic waders – An overview. Wader Study Group Bulletin, 118, 141–152.

[ece37098-bib-0059] Remisiewicz, M. , Bernitz, Z. , Bernitz, H. , Burman, M. S. , Raijmakers, J. M. H. , Raijmakers, J. H. F. A. , Underhill, L. G. , Roskowska, A. , Barshep, Y. , Soloviev, S. A. , & Siwek, I. (2018). Contrasting strategies for wing‐moult and pre‐migratory fuelling in western and eastern populations of Common Whitethroat *Sylvia communis* . Ibis, 161, 824–838.

[ece37098-bib-0060] Rohwer, S. , Viggiano, A. , & Marzhiff, J. M. (2011). Reciprocal tradeoffs between molt and breeding in Albatrosses. Condor, 113, 61–70.

[ece37098-bib-0061] Round, P. D. , & Rumsey, S. (2003). Habitat use, moult and biometrics in the Manchurian Reed Warbler *Acrocephalus tangorum* in Thailand. Ringing & Migration, 21, 215–221.

[ece37098-bib-0062] Rubolini, D. , Massi, A. , & Spina, F. (2002). Replacement of body feathers is associated with low pre‐migratory energy stores in a long‐distance migratory bird, the barn swallow (*Hirundo rustica*). Journal of Zoology, 258, 441–447.

[ece37098-bib-0063] Sander, M. M. , Eccard, J. A. , & Heim, W. (2017). Flight range estimation of migrant Yellow‐browed Warblers *Phylloscopus inornatus* on the East Asian Flyway. Bird Study, 64(4), 569–572.

[ece37098-bib-0064] Sander, M. M. , Heim, W. , & Schmaljohann, H. (2019). Seasonal and diurnal increases in energy stores in migratory warblers at an autumn stopover site along the Asian‐Australasian flyway. Journal of Ornithology, 161, 73–87.

[ece37098-bib-0065] Schaub, M. , & Jenni, L. (2000a). Fuel deposition of three passerine bird species along migration route. Oecologia, 122, 306–317. 28308281 10.1007/s004420050036

[ece37098-bib-0066] Schaub, M. , & Jenni, L. (2000b). Body mass of six long‐distance migrant passerine species along autumn migration route. Journal of Ornithology, 141, 441–460.

[ece37098-bib-0067] Serra, L. , Griggio, M. , Liecheri, D. , & Pilastro, A. (2007). Moult speed constrains the expression of a carotenoid‐based sexual ornament. Journal of Evolutionary Biology, 20, 2038–2043. 10.1111/j.1420-9101.2007.01360.x 17714319

[ece37098-bib-0068] Sleptsov, Y. A. (2018). Data on Nesting Biology of the Pallas´s Grasshopper Warbler *Locustella certhiola rubescens* Blyth, 1845 in Central Yakutia. Bulletin of the Northeast Scientific Center of the Far Eastern Branch of the Russian Academy of Sciences, 3, 87–95 [in Russian].

[ece37098-bib-0069] Stein, H. (2012). Zur Vollmauser eines Rohrschwirls *Locustella luscinoides* . Berichte der Vogelwarte Hiddensee, 21, 81–84.

[ece37098-bib-0070] Steiner, H. M. (1970). Die vom Schema der Passeres abweichende Handschwingenmauser des Rohrschwirls *Locustella luscinoides* . Journal Für Ornithologie, 111, 230–236.

[ece37098-bib-0071] Stepniewska, K. , Ozarowska, A. , Busse, P. , Zehtindjiev, P. , Ilieva, M. , Hnatyna, O. , & Meissner, W. (2018). Fuelling strategies differ among juvenile Sedge and Reed Warblers along the eastern European flyway during autumn migration. Ornis Fennica, 95, 103–114.

[ece37098-bib-0072] Stresemann, E. , & Stresemann, V. (1966). Die mauser der vögel. Journal of Ornithology, 107, 1–448.

[ece37098-bib-0073] Stresemann, E. , & Stresemann, V. (1972). Die postnuptiale und die praenuptiale Vollmauser von *Pericrocotus divaricatus* Raffles. Journal of Ornithology, 113, 435–439.

[ece37098-bib-0074] Svensson, L. (1992). Identification guide to European passerines (4th ed.). Svensson.

[ece37098-bib-0075] Swaddle, J. P. , & Winter, M. S. (1997). The effects of moult on flight performance, body mass, and behaviour of European starlings (*Sturnus vulgaris*): An experimental approach. Canadian Journal of Zoology, 75, 1135–1146.

[ece37098-bib-0076] Thomas, D. K. (1977). Wing moult in the Savi’s Warbler. Ringing & Migration, I, 125–130.

[ece37098-bib-0077] Tonra, C. M. , & Reudink, M. W. (2018). Expanding the traditional definition of molt‐migration. The Auk, 135, 1123–1132.

[ece37098-bib-0078] Underhill, L. G. , & Zucchini, W. (1988). A model for avian primary moult. Ibis, 130, 358–372.

[ece37098-bib-0079] Wickham, H. (2007). Reshaping data with the reshape package. Journal of Statistical Software, 21, 1–20.

[ece37098-bib-0080] Wickham, H. (2016). ggplot2: Elegant graphics for data analysis. Springer.

[ece37098-bib-0081] Williamson, K. (1976). Identification for ringers. 1. The genera Cettia, Locustella, Acrocephalus and Hippolais. Rev.edition. BTO.

[ece37098-bib-0082] Wingfield, J. C. (2005). Flexibility in annual cycles of birds: Implications for endocrine control mechanisms. Journal of Ornithology, 146, 291–304.

[ece37098-bib-0083] Yong, D. L. , Liu, Y. , Low, B. W. , Espanola, C. P. , Choi, C. , & Kawakami, K. (2015). Migratory songbirds in the East‐Asian‐Australasian Flyway: A review from a conservation perspective. Bird Conservation International, 25, 1–37.

