# Supplementary material for: Complex postbreeding molt strategies in a songbird migrating along the East Asian Flyway, the Pallas’s Grasshopper Warbler Locustella certhiola
Source: Ecol Evol. 2020 Dec 21;11(1):11–21. doi: 10.1002/ece3.7098 (PMC7790613; doi:10.1002/ece3.7098)
Supplement: Supplementary file 1 — Supplementary Material [file ECE3-11-11-s001.docx]

Additional Supporting Information:

**Diverse postbreeding molt strategies in a songbird migrating along the East Asian flyway, the Pallas’s Grasshopper Warbler *Locustella certhiola***

**Figure S1.** Map showing the seasonal age distribution of Pallas’s Grasshopper Warblers

between 2011 and 2017 (28 May-21 September) at Muraviovka Park/ Russian Far East.

**Figure S2.** Mean weight of moulting and not moulting Pallas’s Grasshopper Warblers between 2011 and 2017 (28 May-21 September) at Muraviovka Park/ Russian Far East.

**Table S1**. Mean weight of not moulting and moulting Pallas’s Grasshopper Warblers between 28 May and 21 September in 2011-2017 at Muraviovka Park/ Russian Far East.

**Table S2**. Fat score of adult Pallas’s Grasshopper Warblers between 2011 and 2017 (28 May-21 September) at Muraviovka Park/ Russian Far East.

**Table S3**. Muscle score of Pallas’s Grasshopper Warblers between 2011 and 2017 (28 May-21 September) at Muraviovka Park/ Russian Far East.

*
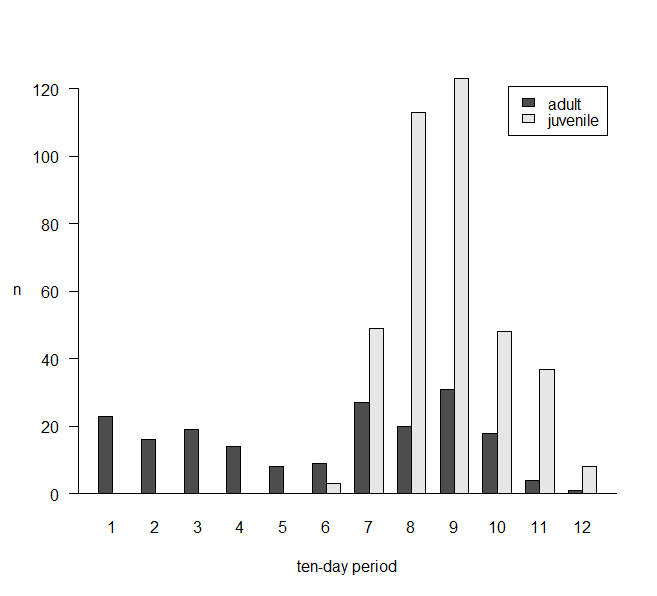
*

**Figure S1**. Pallas’s Grasshopper Warbler seasonal age distribution 2011-2017 at Muraviovka Park/Russian Far East. Numbers of birds caught are presented in dark grey for adult birds and in light grey for juvenile birds. If a bird was captured more than once, only the first handling was considered (i.e., retraps are excluded). Total N = 571; N_juvenile_ = 381; N_adult_ = 190. The first ten-day period starts with the day of the first handling (28th May) and ends on 6th June, followed by the second ten-day period from 7th June to 16th June. Finally, the twelfth ten-day period spans the period from 15th to 24th Sept. The latest handling, however, already took place on the 21st September.


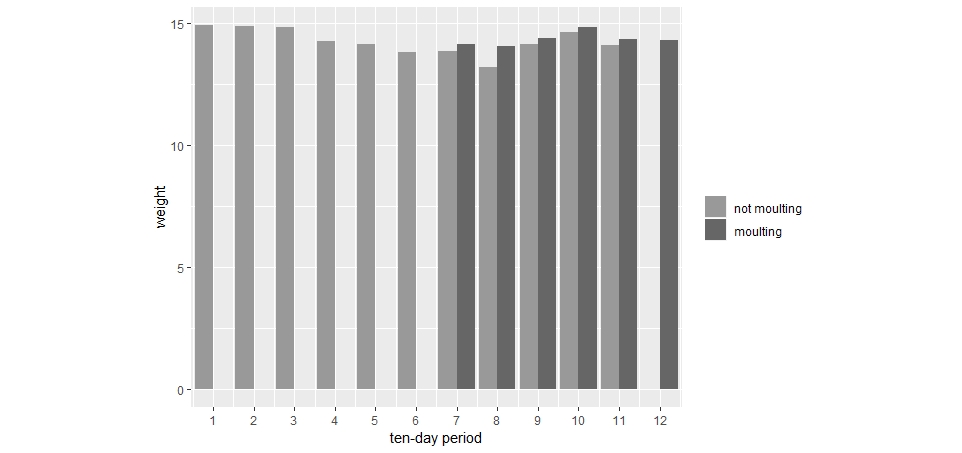


**Figure S2**. Mean weight of moulting and not moulting Pallas’s Grasshopper Warblers between 2011 and 2017 (n = 187) at Muraviovka Park/Russian Far East. The first ten-day period starts with the day of the first handling (28th May) and ends on 6th June, followed by the second ten-day period from 7th June to 16th June. Finally, the twelfth ten-day period spans the period from 15th to 24th Sept.

**Table S1**. Mean weights of not moulting and moulting Pallas’s Grasshopper Warblers (n=187) by ten-day periods between 28 May and 21 September in 2011-2017 at Muraviovka Park/Russian Far East. The first ten-day period starts with the day of the first handling (28th May) and ends on 6th June, followed by the second ten-day period from 7th June to 16th June. Finally, the twelfth ten-day period spans the period from 15th to 24th Sept. Only first handlings of adult birds are considered (mean = arithmetic mean, sd = standard deviation, N_total_ = 187; N_moulting_ = 63; N_not moulting_ = 124).

|  | moulting | | | not moulting | | |
| --- | --- | --- | --- | --- | --- | --- |
| ten-day period | mean | sd | N | mean | sd | N |
| 1 | - | - | 0 | 14.94 | 1.41 | 22 |
| 2 | - | - | 0 | 14.91 | 1.19 | 16 |
| 3 | - | - | 0 | 14.87 | 0.91 | 19 |
| 4 | - | - | 0 | 14.29 | 0.75 | 14 |
| 5 | - | - | 0 | 14.15 | 0.87 | 8 |
| 6 | - | - | 0 | 13.81 | 0.81 | 9 |
| 7 | 14.14 | 0.97 | 13 | 13.87 | 0.99 | 14 |
| 8 | 14.07 | 0.83 | 15 | 13.22 | 0.79 | 5 |
| 9 | 14.41 | 1.11 | 24 | 14.14 | 1.38 | 7 |
| 10 | 14.85 | 1.15 | 8 | 14.67 | 1.21 | 9 |
| 11 | 14.35 | 1.91 | 2 | 14.10 | - | 1 |
| 12 | 14.30 | - | 1 | - | - | 0 |

**Table S2***.* Fat scores of adult Pallas’s Grasshopper Warblers by ten-day periods between 2011 and 2017 at Muraviovka Park/Russian Far East. The first ten-day period starts with the day of the first handling (28th May) and ends on 6th June, followed by the second ten-day period from 7th June to 16th June. Finally, the twelfth ten-day period spans the period from 15th to 24th Sept. Only first handlings of adult birds are considered (mean = arithmetic mean; sd = standard deviation; N_total_ = 189; N_moulting_ = 64; N_not moulting_ = 125).

|  | moulting | | | not moulting | | |
| --- | --- | --- | --- | --- | --- | --- |
| ten-day period | mean | sd | N | mean | sd | N |
| 1 | - | - | 0 | 2.35 | 1.07 | 23 |
| 2 | - | - | 0 | 1.31 | 1.30 | 16 |
| 3 | - | - | 0 | 1.11 | 0.94 | 19 |
| 4 | - | - | 0 | 0.64 | 0.74 | 14 |
| 5 | - | - | 0 | 2.25 | 1.58 | 8 |
| 6 | - | - | 0 | 1.89 | 1.17 | 9 |
| 7 | 1.31 | 0.95 | 13 | 1.64 | 1.15 | 14 |
| 8 | 1.27 | 0.59 | 15 | 1.00 | 0.71 | 5 |
| 9 | 1.38 | 1.10 | 24 | 1.57 | 0.98 | 7 |
| 10 | 2.75 | 1.49 | 8 | 2.22 | 0.97 | 9 |
| 11 | 1.33 | 1.15 | 3 | 0.00 | - | 1 |
| 12 | 1.00 | - | 1 | - | - | 0 |

**Table S3**. Muscle score of adult Pallas’s Grasshopper Warblers by ten-day periods between 2011 and 2017 at Muraviovka Park/Russian Far East. The first ten-day period starts with the day of the first handling (28th May) and ends on 6th June, followed by the second ten-day period from 7th June to 16th June. Finally, the twelfth ten-day period spans the period from 15th to 24th Sept. Only first handlings of adult birds are considered (mean = arithmetic mean; sd = standard deviation; N_total_ = 189; N_moulting_ = 64; N_not moulting_ = 125).

|  | moulting | | | not moulting | | |
| --- | --- | --- | --- | --- | --- | --- |
| ten-day period | mean | sd | N | mean | sd | N |
| 1 | - | - | 0 | 2.70 | 0.47 | 23 |
| 2 | - | - | 0 | 2.56 | 0.51 | 16 |
| 3 | - | - | 0 | 2.21 | 0.42 | 19 |
| 4 | - | - | 0 | 2.00 | 0.39 | 14 |
| 5 | - | - | 0 | 2.50 | 0.53 | 8 |
| 6 | - | - | 0 | 2.11 | 0.33 | 9 |
| 7 | 2.08 | 0.49 | 13 | 2.21 | 0.43 | 14 |
| 8 | 1.93 | 0.26 | 15 | 2.20 | 0.84 | 5 |
| 9 | 2.04 | 0.20 | 24 | 2.00 | 0.00 | 7 |
| 10 | 2.13 | 0.35 | 8 | 2.44 | 0.53 | 9 |
| 11 | 1.67 | 0.58 | 3 | 2.00 | - | 1 |
| 12 | 3.00 | - | 1 | - | - | 0 |
